# Prevalence of immunity to toxoplasmosis among Iranian childbearing age women: Systematic review and meta-analysis

**Published:** 2013-11

**Authors:** Sedigheh Borna, Mamak Shariat, Mohaddese Fallahi, Leila Janani

**Affiliations:** 1 *Maternal-Fetal and Neonatal Research Center, Tehran University of Medical Sciences, Tehran, Iran.*; 2 *Breast Feeding Research Center, Tehran University of Medical Sciences, Tehran, Iran.*; 3 *Department of Epidemiology and Biostatistics, School of Public Health, Tehran University of Medical Sciences, Tehran, Iran.*; 4 *Clinical Trial Center, Tehran University of Medical Sciences, Tehran, Iran.*

**Keywords:** *Immunity*, *Toxoplasmosis*, *Reproductive age women*

## Abstract

**Background:** Our information regarding immunity to toxoplasmosis among reproductive age women is indeterminate and there is significant variation between reported results; it is necessary to perform a Meta-analysis study on subjects to obtain required findings and develop preventive measures accordingly.

**Objective: **Estimation level of immunity to toxoplasmosis in reproductive ages.

**Materials and Methods:** All published papers in main national and international databases were systematically searched for some specific keywords to find the related studies up to 2012. We selected only original articles that either reported percentage of positive anti toxoplasma IgG or total anti toxoplasma antibody by using ELISA or IFAT method (provided that the titer ≥1.20 is considered positive for IFAT) in childbearing age women.

**Results: **Studies involved a total of 13480 participants. The maximum and minimum reported prevalence rates of anti-toxoplasma IgG antibody using IFTA serological method were 21.8% and 54%; and using ELISA serological method were 23% and 64%, respectively. The overall estimation for prevalence of anti-toxoplasma IgG antibody using IFTA serological method was 34.5% (95% CI: 28.5-40.5); and using ELISA method was 37.6% (95% CI: 30.4-44.9). The overall estimation for prevalence of anti-toxoplasma total antibody was 39.9% (95% CI: 26.1-53.7).

**Conclusion:** In Iran, screening of toxoplasma is not routinely performed yet, while the incidence of toxoplasmosis is too high to justify routine screening. Prenatal screening can help to identify mothers susceptible to infection. Screening for the presence of antibodies allows primary prevention of toxoplasmosis infection where eating habits and hygiene practices have clearly been identified as risk factors.

## Introduction

Toxoplasmosis is one of the most widespread infections in animals and humans. It is caused by an intracellular obligatory parasite, Toxoplasma Gondii (T. gondii), which usually transmits to human orally (by ingesting food or water contaminated with oocytes from infected cat feces or tissue cysts in meat). However, blood or leukocyte infusion, organ transplantation and transmission via the placenta are other possibilities of infection (1-3). 

The importance of toxoplasmosis mainly lies among pregnant women (due to the risk of transmission to fetus), transplant patients and immune compromised individuals (4, 5). The incidence of maternal infection during pregnancy is 1 to 8 per 1000 pregnancies. Based on gestational age at seroconversion, risk of transmission and severity of fetal illness are very different. Manifestations of congenital infection are different; even death, but central nervous system and ophthalmic lesions are more common (2, 6). Since these lesions such as severe mental retardation and blindness could be associated with disability, decreased quality of life and increased socioeconomic cost, women in reproductive age are the most important group to call to attention (7).

There are lots of rather limited epidemiological studies that have estimated the prevalence of immunity to toxoplasmosis in reproductive age (childbearing age) women in some provinces of Iran. In addition, the rate of immunity prevalence varies widely from 4.6-74.6% (3, 8). This might be due to the variation in the target population, sampling method, types of laboratory tests and tools, cut off point for positive test (positive test definition), etc. (9). However, comprehensive epidemiological information is required to assess the health significance of this common parasitic infection in any society, based on which, to determine prevalence, severity and the existing risk on population of childbearing age women (10).

"Employing systematic reviews and meta-analysis for identification and analysis of findings from observational studies, one will synthesize research results that are needed by health care professionals and policy makers to provide them with important information on epidemiological indicators (e.g. rate of prevalence and incidence, etc.). Additionally, in meta-analysis sample size is increased as studies are combined, resulting in a better and stronger statistical basis. Meta-analysis can also explore the observed heterogeneity within the results of individual studies" (11).

Since our information regarding immunity to toxoplasmosis among reproductive age women is indeterminate and there is significant variation between reported results; it is necessary to perform a Meta-analysis study on subjects to obtain required findings and develop preventive measures accordingly.

## Materials and methods

**Search strategy**

In this meta-analysis study we searched all probable keyword combinations on Medline/ PubMed and Scopus/ Ovid in order to find relevant studies up to 2012. In addition, related Persian keywords were searched on Iran Medex and Scientific Information Database (SID). All below keyword combinations were searched; "Toxoplasmosis" or "Toxoplasma Gondii" and ("pregnant women" or "reproductive age women" or "childbearing age women") or "prevalence" plus" Iran". Moreover, we screened bibliographies of available studies to maximize sensitivity of the search. 

**Selection criteria and quality assessment**

Anti-Toxoplasma antibody type Immunoglobulin G (IgG) titration determines level of immunity to toxoplasmosis. In addition, there are two serological methods-ELISA (Enzyme-linked immunosorbent assay) and IFAT (Indirect Fluorescent Antibody Test)- to investigate presence of anti-toxoplasma IgG and IgM antibodies. Since sensitivity and specificity of methods and laboratory tools are very different, we selected only original articles that either reported percentage of positive anti-toxoplasma IgG or total anti toxoplasma antibody by using ELISA or IFAT method (provided that the titer ≥1.20 is considered positive for IFAT) in childbearing age women and the full text of papers were found too. Studies were excluded if they were not primary studies (e.g. review articles). In addition, studies which were not representative of the general population were excluded i.e., studies conducted on specific subgroups. 

After initial evaluation, two reviewers independently and carefully reviewed all of the full texts and filled out a standard quality assessment checklist with 7 questions concerning the main methodological aspects of descriptive studies for every study. At this step, reviewers compared scores and discussed every point of disagreement. Finally, those articles with total score of more than 5 were accepted to enter this study.

**Data extraction**

The bibliographic data, methodological information and percentage of immunity to toxoplasmosis in IgG and IgM types were extracted from the papers. In some of the papers that the percentage of IgG had not been reported, we estimated it based on the reported total immunity percentage and IgM percentage. 

**Statistical analysis**

The STATA, ver.11 software was used for statistical analyses. The variance of immunity to toxoplasmosis prevalence in each study was computed based on the binomial formula. We used heterogeneity test (Cochran Q (to explore the variation between studies and found significant heterogeneity between the study findings. Hence, the random effect model was used for estimations. Also, we adjusted all findings of the studies employing Bayesian analysis to minimize the random variation between estimations of the studies. 

The point estimations and their 95% confidence intervals (CIs) were computed and showed in forest plots. In each graph, the size of squares presents the weight of each study and lines in both sides of the squares show the 95% confidence interval of the reported point estimates. Some studies used ELISA (Enzyme -linked immunosorbent assay) method to investigate presence of anti-toxoplasma IgG and IgM antibodies and some of them used IFAT (Indirect Fluorescent Antibody Test)- to explore the presence of anti-toxoplasma IgG and IgM antibodies and also some studies reported total (IgG+IgM) antibodies. Hence, we categorized the results of meta-analysis in 3 subgroups:

1. Studies using IFAT method to explore IgG antibody,
2. Studies using ELISA method to explore IgG antibody,
3. Studies reporting presence of total anti- toxoplasma antibodies.

This meta-analysis study was confirmed to be in accordance with medical ethics measure and correct scientific method by the research council and was financially supported too, by Maternal-Fetal and Neonatal Research Center, Tehran University of Medical Sciences.

## Results

We could find 33 papers regarding immunity to toxoplasmosis prevalence that were conducted in Iran. Finally 22 papers out of 33 were selected. Studies involved a total of 13480 participants with individual study size ranging between 200 and 4120. Summary of the included studies are showed in table I. Nine studies reported presence of anti-toxoplasma IgG antibody using IFTA serological method. 

**Figure 1:**
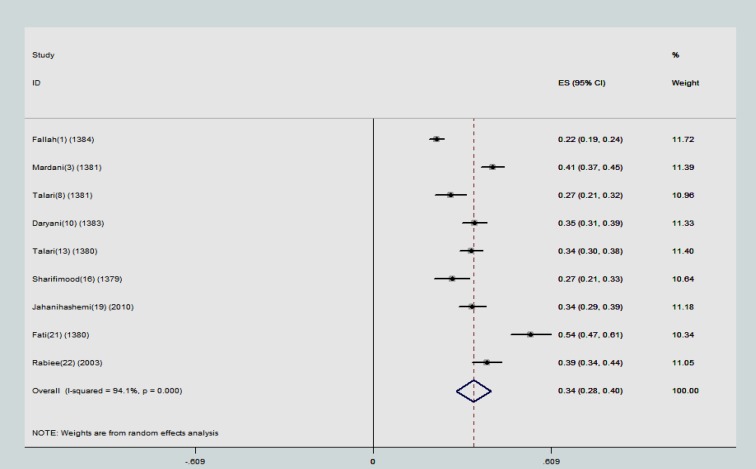
The reported prevalence of anti-toxoplasma IgG antibody using IFTA serological method in different studies. The horizontal lines define the reported 95% confidence interval for the prevalence in each study, and the diamond below the graph shows the pooled prevalence

The minimum prevalence of anti-toxoplasma IgG antibody was reported in Jolfa (21.8%) with a sample size of 1000. The highest rate of prevalence was found in Mashhad (54%). The overall estimation for prevalence of anti-toxoplasma IgG antibody using this serological method was 34.5% (95% CI: 28.5-40.5). The studies in this subgroup had substantial heterogeneity (τ^2^=94.1) (Figure1). Nine studies reported presence of anti-toxoplasma IgG antibody using ELISA serological method. The minimum prevalence of anti-toxoplasma IgG antibody was reported in Bushehr (23.4%). The highest rate of prevalence was reported in Babol (64%) with a sample size of 241individuals.

**Figure 2:**
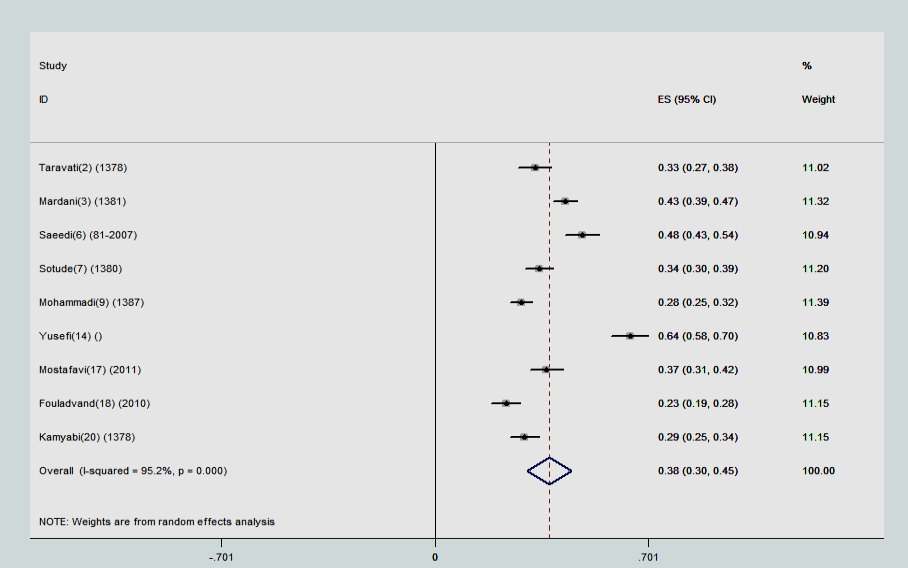
The reported prevalence of anti-toxoplasma IgG antibody using ELISA serological method in different studies. The horizontal lines define the reported 95% confidence interval for the prevalence in each study, and the diamond below the graph shows the pooled prevalence

**Figure 3:**
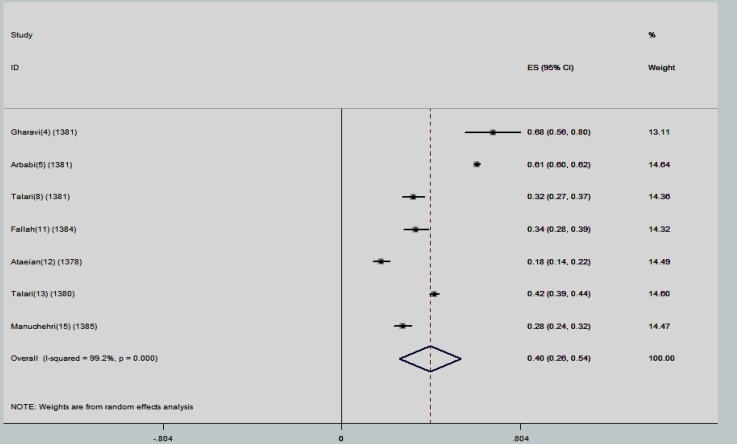
The reported prevalence of anti-toxoplasma total antibody in different studies. The horizontal lines define the reported 95% confidence interval for the prevalence in each study, and the diamond below the graph shows the pooled prevalence

The pooled estimation for prevalence of anti-toxoplasma IgG antibody using ELISA method was 37.6% (95% CI: 30.4-44.9). The studies in this category also had substantial heterogeneity (τ^2^=95.2) (Figure 2). Anti-toxoplasma total antibody was reported in 7 studies. The minimum prevalence of total anti-toxoplasma antibody was reported in Zanjan (17.9%). The highest rate of prevalence was reported in Tehran (68%) with a sample size of 4120. The overall estimation for prevalence of anti-toxoplasma total antibody was 39.9% (95% CI: 26.1-53.7). The studies in this subgroup also had substantial variation (τ^2^=95.2) (Figure 3).

**Table I:** Detailed characteristics of 22 articles included in the systematic review on the prevalence of immunity to toxoplasmosis among Iranian childbearing age women

**Place of study **	**Time of study**	**Language of study**	**Sample size**	**Serological method**	**Immunity prevalence** **(IgG %)**	**Prevalence of infection totally (total Ig %)**
Jolfa (5)	2005	Persian	1000	IFA	21.8	-
Urmia (12)	1999	Persian	300	Elisa	32.8	-
Qom (13)	2002	Persian	600	IFA/Elisa	41/42.8	-
Tehran (6)	2002	Persian	4120	IFA	-	68
Kashan (14)	2002	Persian	340	Elisa	-	61
Gorgan (1)	2002	Persian	300	Elisa	48.3	-
Bandar Abbas (15)	2001	Persian	418	Elisa	34.2	-
Khomeini shahr (16)	2002	Persian	270	IFA	26.6	32.2
Sanandaj (17)	2008	Persian	600	Elisa	28.2	-
Ardebil (18)	2004	Persian	504	IFA	34.7	-
Hamedan (3)	2005	Persian	576	IFA	-	33.5
Zanjan (19)	1999	Persian	1152	IFA	-	17.9
Kashan (20)	2001	Persian	562	IFA	33.7	41.6
Babol (21)	-	Persian	241	Elisa	64	-
Chaharmahal va bakhtiyari (7)	2006	Persian	384	IFA	-	27.6
Zahedan (22)	2000	Persian	200	IFA	27	-
Isfehan (23)	2011	English	300	Elisa	36.6	-
Bushehr (4)	2010	English	303	Elisa	23.4	-
Qazvin (24)	2010	English	400	IFA	34	-
Kerman (25)	1999	Persian	350	Elisa	29.4	-
Mashhad (26)	2001	Persian	200	IFA	54	-
Hamedan (27)	2003	English	360	IFA	38.9	-

## Discussion

T. gondii is a protozoan parasite widely distributed around the world (28). It has been estimated that up to one third of the world's population is infected by T. gondii. Seroprevalence range of T. gondii infection is between 20-90% in different countries. Infections in adults are mostly (90%) asymptomatic. The most common clinical manifestation is cervical lymphadenopathy. Congenital, intrauterine infections cause a wide range of amendments from congenital abnormalities to intrauterine growth deficiencies and fetal death. Acute and latent T. gondii infections during pregnancy are mostly diagnosed by serological tests including detection of anti-T.Gondii-specific IgM and IgG antibodies and avidity of T. gondii-specific IgG antibodies (29).

The epidemiology of toxoplasmosis in many countries has been investigated but a national survey on population of childbearing age women has not been performed in Iran. In the current study, we evaluated seropositivity of 13480 individuals living both in the south and the north of the country.The findings in the present study indicated that Toxoplasma infections are common among population of the Iranian childbearing age women.

The seroprevalence of toxoplasma specific IgG was reported 34.5% by using IFTA serological method and 37.6% by using ELISA serological method in women at childbearing age. The figures clearly vary from 21.8% to 64% among the regions. In this study, we found a 21.8% prevalence of latent T. gondii infection in women of Jolfa City, Iran (5). This prevalence is much lower than those reported in the previous studies of women in other regions of Iran. The highest prevalence had been reported in the north of Iran, Babol and Ghaemshare city (30). In these studies the women between 25-35 yr. of age had the highest toxoplasma positive IgG rates.The prevalence increased by age. The reason might be the increasing risk of exposure with age.

Reports of epidemiological studies indicate that prevalence of T. gondii infection in women at childbearing age varies substantially among countries. For instance, in European countries, prevalence of T. gondii infections in pregnant women varies from 9% to 67% (31). In contrast, in Asian countries, low prevalence of T. gondii infection was found in a Korean study, and a Vietnamese study (0.8% and 11.2%, respectively), while prevalence as high as 41.8-55.4% in pregnant women has been reported in Indian, Malaysian and Nepalese populations (28, 32-37).

The seropositivity of T. gondii in a Turkish study was1.34% for IgM and 24.6% for IgG (38). In Qatar among 823 women of childbearing age, the T. gondii IgG and IgM were 35.1% and 5.2% respectively (39). In a study in Beirut the seroprevalence of IgG T. gondii antibodies were found to be 55 % (40). Toxoplasmosis seropositivity is still high in our population compared to countries such as the Korean study and the Vietnamese (28, 32). Results of this study indicate that 60% of Iranian women in childbearing age are at risk of congenital toxoplasmosis for their fetuses.

Routine screening is currently being discussed in several European countries due to the well proven efficacy of the treatment. In Iran, screening is not routinely performed yet. The current study shows that the incidence of the disease is too high to justify routine screening, but National survey studies are required to determine the incidence of acute toxoplasmosis in Iranian women in childbearing age and pregnancy. 

Ideally, all women of childbearing age should know their serological status before conception. Once the maternal serological status is known, screening for maternal then fetal infection during pregnancy is necessary, as is the availability of adequate in-uterus and postnatal care for the infected infants. Prenatal screening can help to identify mothers susceptible to infection. Screening for the presence of antibodies allows primary prevention of toxoplasmosis infection where eating habits and hygiene practices have clearly been identified as risk factors.
